# Acute cytomegalovirus infection complicated by venous thrombosis: a case report

**DOI:** 10.1186/1476-0711-4-11

**Published:** 2005-08-12

**Authors:** Clarisse Rovery, Brigitte Granel, Philippe Parola, Cédric Foucault, Philippe Brouqui

**Affiliations:** 1 Unité des Rickettsies, UMR6020, Faculté de Médecine, Université de la Méditerranée, Marseille, France; 2 Service de Maladies infectieuses et de Médecine tropicale, Hôpital Nord, Chemin des Bourrelys, 13915 Marseille Cedex 20, France; 3 Service de Médecine Interne, Hôpital Nord, Chemin des Bourrelys, 13915 Marseille Cedex 20, France

## Abstract

**Background:**

CMV-induced vasculopathy and thrombosis have been reported, but they are rare conditions usually encountered in immunocompromised patients. However more and more complications of CMV infections are recognized in immunocompetent patients.

**Case presentation:**

We present a case report of a previously healthy adult with cytomegalovirus infection that was complicated by tibiopopliteal deep venous thrombosis and in whom Factor V Leiden heterozygous mutation was found.

**Conclusion:**

This new case report emphasizes the involvement of cytomegalovirus in induction of vascular thrombosis in patients with predisposing risk factors for thrombosis. It is necessary to screen for CMV infection in patients with spontaneous thrombosis and an history of fever.

## Backgound

CMV-induced vasculopathy and thrombosis have been reported, but they are rare conditions. The few published reports on these conditions focus either on immunocompromised transplant recipients who are receiving high-dose immunosuppressive agents [1,2] or on HIV-infected patients [3-5]. We report one new case of acute CMV infection in a non-immunocompromised adult that presented as spontaneous venous thrombosis. There are few reports involving immunocompetent adults, and these reports are additional arguments for the implication of CMV in vasculopathy and thrombosis.

## Case presentation

A 33-year-old white man with a 5-day history of fever, was hospitalized on May 2004. He also reported epigastralgia and pain in the right leg. He had an history of femoral deep venous thrombosis ten years before. His mother and his brother had an history of deep venous thrombosis and his father died because of pulmonary embolism. Physical examination revealed fever (temperature range 38°C–38°5C), asthenia, headache, anorexia and epigastralgia. His right leg was enlarged and painful. Tibiopopliteal deep venous thrombosis was confirmed by Doppler ultrasonography. At the time of hospital admission, his WBC was 6,000 cells/mm^3^, with 2,100 neutrophils/mm^3 ^and 2,820 lymphocytes/mm^3^, with 9% of hyperbasophilic lymphocytes. His platelet count was 120,000/mm^3 ^and his hemoglobin level was 141 g/l. The C-reactive protein level was 52 mg/l. Liver function tests revealed an ALT level of 65 UI/l, an AST level of 64 UI/l, an LDH level of 891 UI/l, a PAL level of 61 UI/l and a γ-GT level of 41 UI/l. Blood cultures were sterile. The results of serological tests for HIV ELISA, hepatitis A IgM, hepatitis B surface antigen, hepatitis C virus, Q fever, and toxoplasma were negative. VCA and EBNA IgG antibodies were positive suggesting past immunization. Serological test for CMV ELISA was strongly positive for IgM antibodies. The result of a CMV pp65 antigenemia assay, based on the direct detection of the CMV pp65 phosphoprotein was positive. A second serological test for CMV taken two weeks after the first one showed seroconversion with appearance of IgG. No pathological values for prothrombin time ratio, activated partial thromboplastin time, plasma antithrombin III, protein C and S activity were found. Results of tests for anticardiolipin antibodies, lupus anticoagulant and prothrombin 20210 were negative. Antinuclear antibodies were negative and complement was normal. Factor V Leiden heterozygous mutation was found. Anticoagulant treatment was introduced. Tthe patient became asymptomatic and was discharged from the hospital.

## Discussion

Our observation, as well those of others [6,7], argues for implication of CMV in vasculopathy and thrombosis. In these cases, thrombosis occurred in immunocompetent patients during CMV infection, which was confirmed, in most cases, by pp65 antigen and/or CMV viremia/viruria. There are few reports involving immunocompetent adults since we retrieved only 13 reports through MEDLINE database [6-16]. Predisposing risk factors for thrombosis have sometimes been found in the reported cases such as protein C and S deficiency [15] and the presence of antiphospholipid antibodies [10,13,16,17]. In other reports, patients were women and had risk factors such as oral contraceptive use [8,9,14]. The factor V Leiden heterozygous mutation was only reported once previously [6]. However in some other reported cases, no hemostatic abnormalities can be found [6,7,17] and spontaneous resolutions of thrombosis were observed in some of these patients who did not receive anticoagulant treatment [9]. This suggests that CMV was the sole triggering factor. These observations suggest that acute CMV infection may be the cause of the thrombosis event in these patients. However, it is difficult to determine whether CMV is a direct cause of thrombosis or a precipitating factor in patients with underlying thrombogenic tendency. Considering that CMV could be a transient triggering factor for thrombosis in our patient, we decided not to treat lifelong as recommended for patients with factor V Leiden heterozygous mutation and 2 spontaneous thrombosis [18] but only for 6 months [19].

In conclusion, our case report reinforces the opinion that CMV is a rare but potentially signifiant cause or precipitating factor of arterial and venous thrombosis in immunocompetent hosts [20]. It seems to be necessary to screen for CMV infection in patients with spontaneous thrombosis and an history of fever. It is important to recognize CMV infection in these patient as it may be possible to consider the discontinuation of anticoagulant therapy earlier.
